# High infection rates and risk‐adapted prevention strategies in contemporary pediatric allogeneic hematopoietic stem cell transplantation

**DOI:** 10.1002/pdi3.101

**Published:** 2024-07-14

**Authors:** Tsz Wing Yeung, Wilson Yau Ki Chan, Sally Cheuk Ying Wong, Pamela Pui Wah Lee, Daniel Ka Leung Cheuk, Wing Leung

**Affiliations:** ^1^ Department of Paediatrics and Adolescent Medicine Hong Kong Children's Hospital Kowloon Hong Kong; ^2^ Department of Pathology Hong Kong Children's Hospital Kowloon Hong Kong; ^3^ Department of Paediatrics and Adolescent Medicine Queen Mary Hospital Li Ka Shing Faculty of Medicine The University of Hong Kong Pok Fu Lam Hong Kong

**Keywords:** bacterial infections, hematopoietic stem cell transplantation, invasive fungal diseases, pediatric, viral infections

## Abstract

With recent changes in hematopoietic stem cell transplantation (HSCT) practices, such as the increasing use of alternative donors and ex vivo T‐cell depletion, how various risk factors interplay and affect the timeline of infections have not been well elucidated. We retrospectively reviewed the first 100 consecutive HSCT from April 2019 to October 2021 in the only pediatric HSCT center in Hong Kong. We found that the vast majority of the allogeneic transplant recipients (69/74, 93.2%) had infections after HSCT, amongst which bacterial, viral, and fungal infection rates were 35.1%, 90.5%, and 9.5%, respectively. In contrast, only 30.8% (8/26) of autologous transplant recipients had infections (rate of bacterial, viral, and fungal infection were 19.2%, 15.4%, and 3.8%, respectively). Human herpesvirus 6 (HHV‐6) and BK virus (BKV) typically occurred early after HSCT, adenovirus (ADV) and varicella zoster virus (VZV) thereafter, and cytomegalovirus (CMV) and Epstein–Barr virus (EBV) throughout the entire 2.5‐year observation period. Ex vivo T‐cell depletion was a general risk factor for viral infection with HHV‐6 (hazard ratio [HR] = 3.03), BKV (HR = 3.36), CMV (HR = 4.45), and EBV (HR = 7.15); all *p* < 0.02. Cancer in second‐complete remission compared with first‐complete remission was a risk factor for bacterial infection (*OR* = 6.0, 95% *CI* = 1.12–32.2, and *p* = 0.037). Patients with gut graft‐versus‐host disease were at risk for fungal infections (*OR* = 12.3, 95% *CI* = 1.33–114.4, and *p* = 0.027). The infection‐related mortality rate was 10.0%, which occurred only in allogeneic HSCT patients with hematological malignancies receiving cord blood (*n* = 4) or haploidentical HSCT (*n* = 6). Collectively, our findings in pediatric patients after contemporary HSCT support both time‐dependent and risk‐adapted measures against infective complications to improve transplant outcome.

## Graphical abstract

An evidence‐based algorithm for prophylactic antimicrobials and infection monitoring after contemporary pediatric hematopoietic stem cell transplantation.

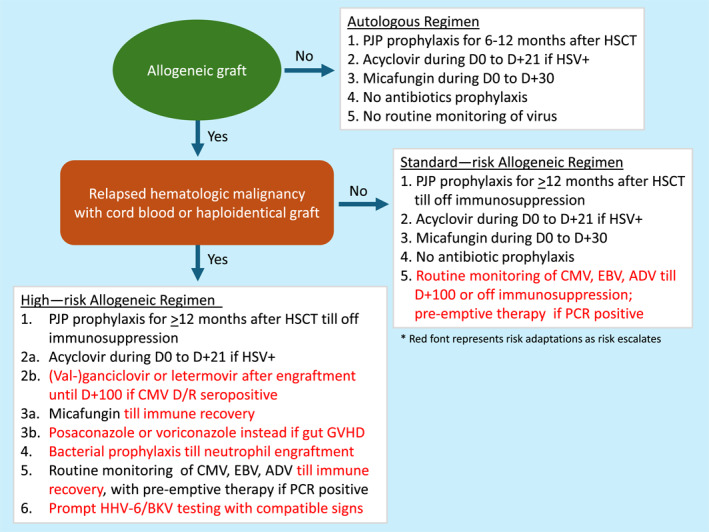

## 1 INTRODUCTION

Hematopoietic stem cell transplantation (HSCT) is widely used for curing malignant and nonmalignant disorders. Infection is a major cause of morbidity and mortality after HSCT.^1^ According to the Center for International Blood and Marrow Transplant Research report in 2020, infections accounted for 16% of deaths after matched‐related donor HSCT, 27% after haploidentical donor HSCT, 14% after unrelated donor HSCT, and 28% after autologous HSCT within 100 days posttransplant in the US pediatric population.^2^ Antimicrobial prophylaxis and routine surveillance with preemptive therapy are therefore important supportive measures.^3^, ^4^, ^5^, ^6^, ^7^, ^8^, ^9^ As severe and fatal infections still occur despite these measures, reexamination of the epidemiology and risk‐adapted measures against infective complications after contemporary HSCT is warranted.

Comprehensive epidemiological studies of bacterial, viral, and fungal infections after conventional pediatric HSCT have been published.^10^, ^11^ Other studies have focused on a particular pathogen,^12^, ^13^, ^14^, ^15^, ^16^, ^17^, ^18^ certain type of HSCT,^16^, ^19^, ^20^ individual primary disease,^21^, ^22^, ^23^ or specific local perspective.^24^, ^25^ Many recent changes in HSCT approach could affect the epidemiology of infections posttransplant. For example, there is an increasing use of cord blood, haploidentical donors, peripheral blood stem cells (PBSCs), and ex vivo T‐cell depletion, all of which could be associated with an increase in infection‐related mortality (IRM) and complications after HSCT.^4^, ^26^ How these factors interplay and affect the timeline and the risk of various infections have not been well elucidated in children. The aim of this study is to describe the epidemiology, risk factors, and outcome of infections after contemporary HSCT to inform future strategies against infective complications.

## 2 METHODS

### 2.1 Setting and patients

The first 100 consecutive allogeneic and autologous HSCTs performed at Hong Kong Children's Hospital since its opening in 2019 were included in this retrospective cohort study. All patients were monitored for a long term after transplantation without loss of follow‐up. Patient data and laboratory results were collected from electronic medical records until December 31, 2021. The study was approved by the local Institutional Review Board.

### 2.2 Definitions of transplant variables

Engraftment is defined as the first of three consecutive days with an absolute neutrophil count ≥0.5 × 10^9^/L. Conditioning chemotherapy regimen that included total body irradiation (TBI) ≥5 Gy in single fraction or ≥ 8 Gy fractionated; busulfan ≥9 mg/kg oral (or intravenous equivalent); melphalan >150 mg/m^2^; and thiotepa ≥10 mg/kg were classified as myeloablative.^27^ Diagnosis of acute and chronic graft‐versus‐host disease (GVHD) were based on clinical criteria.^28^, ^29^

### 2.3 Definitions of infections

Infections were categorized according to the phases of immune recovery posttransplantation: pre‐engraftment phase (day 0–30), post‐engraftment phase (31–100 days), and late post‐engraftment phase (101 days–2 years). Infection occurring during conditioning was included as infection in pre‐engraftment phase. The day of onset of infection was defined as the day of the first positive diagnostic sample. Infections with the same organism occurring more than 14 days after the last negative culture or diagnostic test were recorded as two separate infectious episodes.

### 2.4 Bacterial infection

Bacterial infection was defined as isolation of an organism associated with symptoms or disease. Bacteremia, urinary tract infection, skin and soft tissue infection, and gastrointestinal infection were included, while *Clostridium difficile* gastroenteritis was excluded in the analysis. Colonization detected in surveillance cultures and positive blood cultures due to contamination were not considered as infections.

### 2.5 Viral infection and reactivation

Viral pathogens detected by preemptive screening of peripheral blood samples were included. Cytomegalovirus replication in peripheral blood was monitored twice weekly after allogeneic HSCT till day 100 and once weekly in autologous HSCT within 30 days posttransplant using real‐time polymerase chain reaction (PCR). Patients with detectable CMV DNA were monitored twice weekly. CMV infection was treated with valganciclovir or foscarnet until PCR were negative in 2 consecutive assays if there is no evidence of end‐organ disease. Epstein–Barr virus and adenovirus surveillance were done weekly for allogeneic HSCT by real‐time PCR. PCR for HHV‐6, human HHV‐7, and BKV were done as clinically indicated. The PCR lower limit of quantification was 200 IU/mL for CMV, 150 IU/mL for EBV, 400 copies/mL for ADV, 100 copies/mL for HHV6, and 700 copies/mL for BKV. For VZV, infection diagnosed by either PCR assay or clinical diagnosis was included.

### 2.6 Fungal infection

Fungal surveillance was performed with stool samples on the first‐week posttransplantation then alternate week until engraftment. 1,3‐β‐D‐glucan and galactomannan tests were only performed as clinically indicated. The diagnosis of invasive fungal disease (IFD) was made according to the European Organization for Research and Treatment of Cancer/Mycoses Study Group (EORTC/MSG) criteria^30^ as proven, probable, or possible IFD. Possible IFD was not included in the analysis.

### 2.7 Infection prophylaxis

All patients received prophylaxis for *Pneumocystis jirovecii* until at least 1 year after transplantation and without immunosuppressant. Majority of patients received broad spectrum beta‐lactam antibiotics due to febrile neutropenia before engraftment. From April 2019 to August 2020, patients received fluconazole or micafungin from day 0 to day +30 as antifungal prophylaxis; all received micafungin after August 2020. Patients received valganciclovir or ganciclovir prophylaxis from engraftment to day +100 if recipient and/or donor were CMV seropositive in allogeneic transplants. From late December 2020, a preemptive approach was adopted and no routine anti‐CMV prophylaxis was given. Recipients seropositive for herpes simplex virus (HSV) or had previously documented HSV infection would receive acyclovir prophylaxis from day 0 to day +21.

### 2.8 Definition of infection‐related mortality

IRM was defined as death that occurred in the presence of infection that was severe enough to contribute to death (e.g., bacteremia, viral pneumonia, and disseminated fungal infection), with or without the presence of relapse, GVHD, organ failure, or other comorbidities.

### 2.9 Statistical analysis

Statistical analyses were performed using SPSS version 26 or Stata version 16. Categorical variables were compared by Chi‐squared test or Fisher's exact test where appropriate. For risk factor analyses, dependent variables included type of transplantation, gender, age group, donor and graft source, underlying disease (leukemia and lymphoma, solid tumor, or nonmalignant diseases), conditioning type (TBI or non‐TBI‐based) and intensity (myeloablative or not), any ex vivo αβ T‐cell depletion of graft, history of steroid exposure (use of intravenous methylprednisolone or oral prednisolone after conditioning started) and steroid use for GVHD (including intravenous methylprednisolone, oral prednisolone, and budesonide), history of bacteremia within 6 months before HSCT, presence of acute or chronic GVHD, and CMV serostatus. Time‐dependent covariates, such as GVHD and steroid use for GVHD, were coded as positive if their onset occurred before the infection event. Hazard functions were compared using the log‐rank test; factors with *p* value <0.05 were included in multivariate analysis with Cox regression models. Relative odds were compared using logistic regression. A *p* value <0.05 was considered statistically significant.

## 3 RESULTS

### 3.1 Transplant characteristics

A total of 86 patients underwent 100 HSCT within the 30‐month study period; 26 transplants were autologous and 74 were allogeneic. Their clinical and transplant characteristics are summarized in Table 1. The median follow‐up duration was 292 days (range 5–974) for the entire cohort and 331.5 days (range 25–974) for survivors.

**TABLE 1:** Clinical and transplant characteristics.

	Allogeneic, *n* = 74	Autologous, *n* = 26
Gender, *n* (%)		
Male	49 (66.2)	13 (50.0)
Female	25 (33.8)	13 (50.0)
Median age (range)	10.2 (0.1–18.2)	2.8 (1.4–11.6)
Age, *n* (%)		
0 ≤ 3	11 (14.9)	16 (61.5)
3 ≤ 11	33 (44.6)	9 (34.6)
11 ≤ 19	30 (40.5)	1 (3.8)
Race, *n* (%)		
Chinese	69 (93.2)	23 (88.5)
Non‐Chinese	5 (6.8)	3 (11.5)
Underlying disease, *n* (%)		
ALL	20 (27.0)	0 (0)
AML	18 (24.3)	0 (0)
CML	1 (1.4)	0 (0)
JMML	1 (1.4)	0 (0)
MDS	5 (6.7)	0 (0)
Lymphoma	3 (4.0)	0 (0)
Solid tumor	4 (5.4)	26 (100)
Hematological disorders	17 (23.0)	0 (0)
Immunological disorders	1 (1.4)	0 (0)
Metabolic disorders	4 (5.4)	0 (0)
Number of transplant, *n* (%)		
1st	60 (81.0)	19 (73.1)
2nd	13 (17.6)	4 (15.4)
3rd	1 (1.4)	3 (11.5)
Product type, *n* (%)		
Peripheral blood stem cell	48 (64.8)	25 (96.2)
Bone marrow	11 (14.9)	0 (0)
Umbilical cord blood	15 (20.3)	1 (3.8)
Donor type, *n* (%)		
Matched unrelated donor	11 (14.9)	‐
Matched sibling donor	10 (13.5)	‐
Haploidentical donor	38 (51.3)	‐
Umbilical cord blood	15 (20.3)	‐
CMV D/R status, *n* (%)		
D+/R+	31 (41.9)	‐
D+/R−	17 (23.0)	‐
D−/R+	13 (17.6)	‐
D−/R−	9 (12.1)	‐
D unknown/R+	4 (5.4)	‐
Conditioning, *n* (%)		
TBI‐based	22 (29.7)	0 (0)
Non‐TBI‐based	52 (70.3)	26 (100)
Myeloablative	56 (75.7)	25 (96.2)
Non‐myeloablative	18 (24.3)	1 (3.8)
T‐cell depletion, *n* (%)		
Yes	24 (32.4)	‐
No	50 (67.6)	‐
Median time to engraft (range)	13 (9–35)	12 (10–31)
Time to engraft, *n* (%)		
Within 28 days	69 (93.2)	24 (92.3)
>28 days	1 (1.4)	2 (7.7)
Failure to engraft	4 (5.4)	0 (0)
GVHD, *n* (%)		
Acute	44 (59.5)	‐
Chronic	24 (32.4)	‐

Abbreviations: ALL, acute lymphoblastic leukemia; AML, acute myeloid leukemia; CML, chronic myeloid leukemia; CMV D/R, cytomegalovirus donor/recipient; GVHD, graft‐versus‐host disease; JMML, juvenile myelomonocytic leukemia; MDS, myelodysplastic syndrome; RIC, reduced‐intensity conditioning; TBI, total body irradiation.

### 3.2 Overall incidence of infection

Only 6.8% (5/74) of allogeneic transplants did not have any bacterial, viral, or fungal infection after the procedure, in contrast to an infection‐free rate of 69.2% (18/26) after autologous transplantations.

### 3.3 Bacterial infection

#### 3.3.1 Incidence

The incidence of bacterial infections was 35.1% (26/74) after allogeneic HSCT and 19.2% (5/26) after autologous HSCT. The frequencies of gram‐positive (GP) and gram‐negative (GN) bacterial infections were 14.9% (11/74) and 27.0% (20/74), respectively, in allogeneic transplants, and 3.8% (1/26) and 15.4% (4/26), respectively, in autologous transplants.

#### 3.3.2 Timing

**FIGURE 1:**
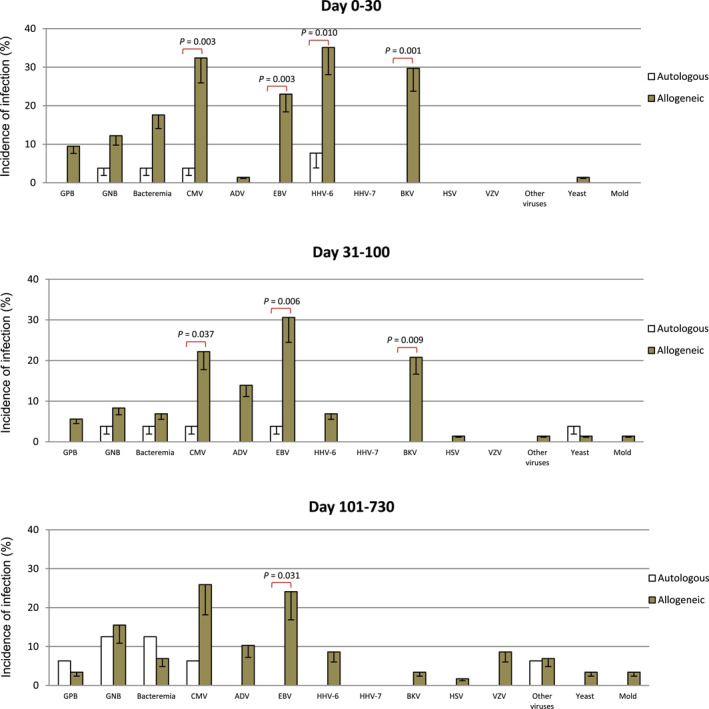
Incidences of infections between 0 and 30 days, 31 and 100 days, and 101 days and 2 years after autologous and allogeneic transplantations. ADV, adenovirus; BKV, BK virus; CMV, cytomegalovirus; EBV, Epstein‐Barr virus; GNB, Gram‐negative bacteria; GPB, Gram‐positive bacteria; HHV‐6, human herpesvirus 6; HHV‐7, human herpesvirus 7; HSV, herpes simplex virus; VZV, varicella zoster virus.

Figure 1 shows the episodes of GP and GN bacterial infections and bacteremia in specified time periods posttransplantation. Their frequencies were similar in the 3 time periods among both autologous and allogeneic recipients.

#### 3.3.3 Bacteremia

In allogeneic transplants, there were 22 episodes of bacteremia (Supplemental Table S1), with a median onset time of 12 days (range 8–248) and a similar distribution of GP and GN organisms (45.5% vs. 54.5%). Among GP bacteremia, coagulase‐negative *staphylococci,* including *Staphylococcus haemolyticus* and *Staphylococcus epidermidis*, were the most frequent pathogen isolated; while *Pseudomonas aeruginosa* and *Enterobacter cloacae* were the most frequent GN pathogens. In autologous transplants, there were 4 bacteremia with a median onset time of 115 days (range 5–147), of which 1 was GP and 3 were GN bacterial infections (including 1 polymicrobial GN bacterial infection).

#### 3.3.4 Risk factors

In univariate analysis, the risk of bacterial infection was higher with the use of cord blood (*p* = 0.041 vs. bone marrow [BM]) or in patients with cancer in second complete remission (CR2) (*p* = 0.011 vs. first complete remission [CR1]). Bacterial infection was not observed in any patients receiving a matched sibling graft (*p* = 0.01 vs. other donor sources). In multivariate analyses, cancer in CR2 remained a risk factor (OR = 6.0, 95% CI = 1.12–32.2, and *p* = 0.037).

### 3.4 Viral infection

#### 3.4.1 Incidence

The cumulative incidence of viremia with any virus within the 30‐month study period was 15.4% (4/26) in autologous HSCT and 90.5% (67/74) in allogeneic HSCT. The frequency among allogeneic transplant recipients was 47.3% (35/74) for CMV, 21.6% (16/74) for ADV, 47.3% (35/74) for EBV, 37.8% (28/74) for HHV‐6, and 41.9% (31/74) for BKV with a median onset time of 25 days (range 0–112), 82.5 days (range 4–163), 32 days (range 4–168), 17 days (range 6–175), and 17 days (range 2–142), respectively. The frequency of VZV reactivation in allogeneic transplants was 6.8% (5/74) and all occurred in 101–730 days posttransplant.

#### 3.4.2 Timing

Comparing the 3 time periods post‐allogeneic transplants, HHV‐6 and BKV were more common in 0–30 days (*p* < 0.001; *p* = 0.001, respectively), ADV was more common between 31 and 100 days (*p* = 0.019), and VZV and other viruses were more common in 101–730 days (*p* = 0.002 and 0.03, respectively) (Supplemental Table S2).

The frequency of viral infection was higher in allogeneic than autologous transplants in all 3 periods posttransplant (71.6% vs. 11.5% in 0–30 days, *p* < 0.001; 58.3% vs. 3.8% in 31–100 days, *p* < 0.01; 53.4% vs. 12.5% in 101–730 days, *p* = 0.004). Compared with autologous transplants, the frequencies of CMV reactivation in 0–30 days (32.4% vs. 3.8%, *p* = 0.003) and 31–100 days (22.2% vs. 3.8%, *p* = 0.037), EBV in all 3 time period posttransplant (24.3% vs. 0% in 0–30 days, *p* = 0.003; 29.2% vs. 3.8% in 31–100 days, *p* = 0.006; 24.1% vs. 0% in 101–730 days, *p* = 0.031), HHV‐6 in 0–30 days (35.1% vs. 7.7%, *p* = 0.01), and BKV in 0–30 days (29.7% vs. 0%, *p* = 0.001) and 31–100 days (20.8% vs. 0%, *p* = 0.009) were significantly higher in allogeneic transplants (Figure 1).

#### 3.4.3 Severe viral disease

In allogeneic transplants, seven patients had CMV pneumonia and 1 had CMV colitis. Three patients had ADV pneumonia and 4 had ADV enteritis. One patient had probable EBV‐related posttransplant lymphoproliferative disease (EBV‐PTLD) presented with lymphadenopathy and hepatosplenomegaly, and 2 had proven EBV‐PTLD. One patient had HHV‐6 encephalitis and 1 had HHV‐6 pneumonia. In patients with BK viruria, 11 had hemorrhagic cystitis. Among 5 patients with VZV reactivation, 1 had disseminated zoster. There was no severe viral disease in autologous HSCT.

#### 3.4.4 Risk factors

##### 3.4.4.1 CMV

In univariate analysis, the use of alternative donor transplants (*p* = 0.0008 vs. matched sibling donor [MSD] and autologous), relapsed/refractory cancer status (*p* = 0.002 vs. CR1), Donor+/Recipient+ (D+R+) CMV serostatus (*p* < 0.0001), T‐cell depletion (*p* = 0.0052 vs. T‐cell replete), and steroid exposure (*p* = 0.0002 vs. no steroid exposure) were associated with a higher risk of CMV reactivation (Supplemental Figure S1). Multivariate analysis confirmed that refractory disease versus CR1 (HR = 8.76, 95% CI = 1.49–51.4, and *p* = 0.016), D+R+ versus D+R− (HR = 16.77, 95% CI = 2.41–116.66, and *p* = 0.004) and D−R+ versus D+R− CMV status (HR = 7.7, 95% CI = 1.11–53.39, and *p* = 0.039), T‐cell depletion (HR = 4.45, 95% CI = 1.34–14.75, and *p* = 0.015), and steroid exposure (HR = 13.31, 95% CI = 2.15–82.35, and *p* = 0.005) (Table 2) had a higher risk for CMV reactivation.

**TABLE 2:** Multivariate analysis of factors affecting the risk for viral infections.

Virus	Risk factor	HR (95% CI)	*p* value
CMV	Refractory disease versus CR1	8.76 (1.49–51.40)	0.016
	CMV D/R status: D+R+ versus D+R−	16.77 (2.41–116.66)	0.004
	CMV D/R status: D−R+ versus D+R−	7.70 (1.11–53.39)	0.039
	T‐cell depletion versus no depletion	4.45 (1.34–14.75)	0.015
	Steroid exposure versus no exposure	13.31 (2.15–82.35)	0.005
ADV	Age group 11–18 versus 0–10	3.19 (1.13–9.07)	0.029
	TBI‐based conditioning versus non‐TBI‐based	3.64 (1.28–10.36)	0.015
	Steroid exposure versus no exposure	8.33 (1.06–65.62)	0.044
	Bacteremia 6 months prior to HSCT versus no bacteremia	4.49 (1.54–13.08)	0.006
EBV	Haploidentical versus matched sibling donor	0.18 (0.04–0.86)	0.031
	Umbilical cord blood versus matched sibling donor	0.25 (0.06–0.97)	0.045
	Autologous versus matched sibling donor	0.05 (0.01–0.38)	0.004
	T‐cell depletion versus no depletion	7.15 (1.62–31.58)	0.009
HHV‐6	T‐cell depletion versus no depletion	4.28 (1.49–12.28)	0.007
	Steroid for GVHD versus no steroid	3.03 (1.35–6.82)	0.007
BKV	Age group 3–10 versus 11–18	0.43 (0.20–0.92)	0.030
	Umbilical cord blood versus bone marrow	20.83 (2.56–169.31)	0.005
	T‐cell depletion versus no depletion	3.36 (1.34–8.45)	0.010

Abbreviations: ADV, adenovirus; BKV, BK virus; CI, confidence interval; CMV, cytomegalovirus; CMV D/R, cytomegalovirus donor/recipient; CR1, first complete remission; EBV, Epstein–Barr virus; GVHD, graft‐versus‐host disease; HHV‐6, human herpesvirus 6; HR, hazard ratio; HSCT, hematopoietic stem cell transplantation; TBI, total body irradiation.

##### 3.4.4.2 ADV

**FIGURE 2:**
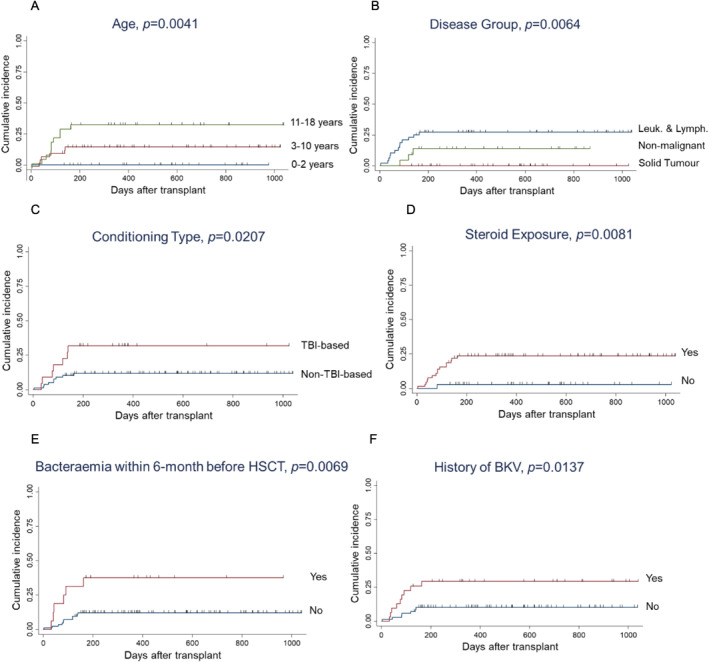
Cumulative incidences of adenovirus. (A–F) Stratification based on various risk factors. BKV, BK virus; HSCT, hematopoietic stem cell transplantation; Leuk. and Lymph., leukemia and lymphoma; TBI, total body irradiation.

Univariate analysis revealed that patients in the age group of 11–18 years (*p* = 0.0041 vs. younger age groups), with underlying leukemia or lymphoma (*p* = 0.0064 vs. solid tumor and nonmalignant diseases), TBI‐based conditioning (*p* = 0.0207), and history of steroid exposure (*p* = 0.0081) were associated with a higher risk for ADV (Figure 2). Patients with history of bacteremia within 6 months before HSCT (*p* = 0.0069) or BKV infection after HSCT (*p* = 0.0137) were also significantly associated with ADV infection/reactivation. Older age group 11–18 versus 0–10 years (HR = 3.19, 95% CI = 1.13–9.07, and *p* = 0.029), TBI‐based conditioning (HR = 3.64, 95% CI = 1.28–10.36, and *p* = 0.015), steroid exposure (HR = 8.33, 95% CI = 1.06–65.62, and *p* = 0.044), and history of bacteremia within 6 months prior to HSCT (HR = 4.49, 95% CI = 1.54–13.08, and *p* = 0.006) remained significant risk factors in multivariate analysis (Table 2).

##### 3.4.4.3 EBV

In univariate analysis, older age group (*p* = 0.0054), MSD compared with other donor sources (*p* = 0.0004), TBI‐based conditioning (*p* = 0.0004), T‐cell depleted graft (*p* < 0.0001), and history of CMV viremia (*p* = 0.0061) predicted EBV reactivation (Supplemental Figure S2). Multivariate analysis confirmed the findings that haploidentical HSCT (HR = 0.18, 95% CI = 0.04–0.86, and *p* = 0.031), umbilical cord blood (UCB) HSCT (HR = 0.25, 95% CI = 0.06–0.97, and *p* = 0.045), and autologous HSCT (HR = 0.05, 95% CI = 0.01–0.38, and *p* = 0.004) were associated with a lower risk of EBV reactivation compared with MSD. Multivariate analysis also showed significant risk in HSCT with T‐cell depleted graft (HR = 7.15, 95% CI = 1.62–31.58, and *p* = 0.009) (Table 2).

##### 3.4.4.4 HHV‐6

**FIGURE 3:**
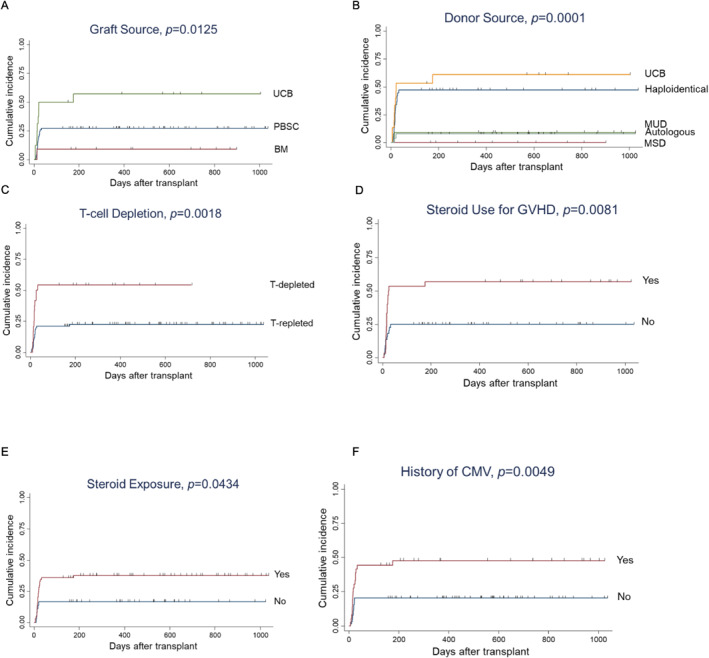
Cumulative incidences of human herpesvirus 6. (A–F) Stratification based on various risk factors. BM, bone marrow; CMV, cytomegalovirus; GVHD, graft‐versus‐host disease; MSD, matched sibling donor; MUD, matched unrelated donor; PBSC, peripheral blood stem cell; UCB, umbilical cord blood.

Univariate analysis suggested UCB versus other graft source (*p* = 0.0125 vs. PBSC and BM), UCB and haploidentical HSCT versus other donor sources (*p* = 0.0001), T‐cell depleted graft (*p* = 0.0018), steroid use for GVHD (*p* = 0.0081), steroid exposure (*p* = 0.0434), and CMV viremia (*p* = 0.0049) were associated with a higher risk for HHV‐6 viremia (Figure 3). While in multivariate analysis, T‐cell depletion (HR = 4.28, 95% CI = 1.49–12.28, and *p* = 0.007) and steroid use for GVHD (HR = 3.03, 95% CI = 1.35–6.82, and *p* = 0.007) were significant risk factors (Table 2).

##### 3.4.4.5 BKV

**FIGURE 4:**
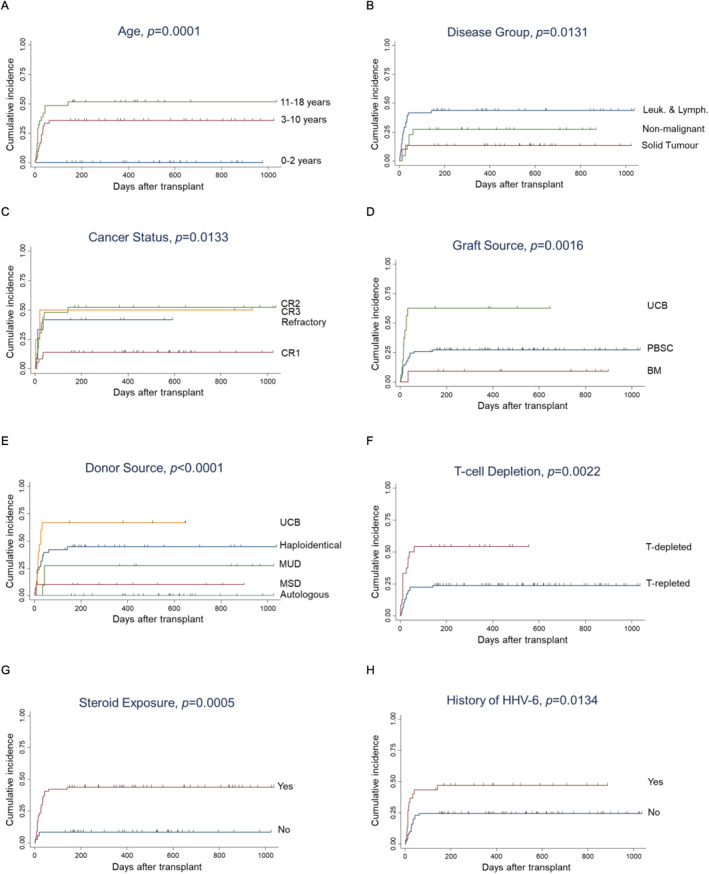
Cumulative incidences of BK virus. (A–H) Stratification based on various risk factors. BM, bone marrow; CR1, first complete remission; CR2, second complete remission; CR3, third complete remission; HHV‐6, human herpesvirus 6; Leuk. and Lymph., leukemia and lymphoma; MSD, matched sibling donor; MUD, matched unrelated donor; PBSC, peripheral blood stem cell; UCB, umbilical cord blood.

In univariate analysis, older age group (*p* = 0.0001), underlying leukemia or lymphoma (*p* = 0.0131 vs. solid tumor and nonmalignant diseases), refractory disease (*p* = 0.0133 vs. CR1), UCB versus other graft sources (*p* = 0.0016 vs. PBSC and BM) or other donor sources (*p* < 0.0001), T‐cell depleted graft (*p* = 0.0022), steroid exposure (*p* = 0.0005), and history of HHV‐6 viremia (*p* = 0.0134) were predictive of BKV reactivation (Figure 4). UCB versus marrow graft (HR = 20.83, 95% CI = 2.56–169.31, and *p* = 0.005) and T‐cell depletion (HR = 3.36, 95% CI = 1.34–8.45, and *p* = 0.01) remained risk factors for BKV in multivariate comparison (Table 2). Lower age group 3–10 (vs. 11–18 years) was associated with less BKV reactivation (HR = 0.43, 95% CI = 0.20–0.92, and *p* = 0.03).

### 3.5 Fungal infection

#### 3.5.1 Incidence

The cumulative incidence of IFD within the 30‐month study period was 9.5% (7/74) in allogeneic and 3.8% (1/26) in autologous transplants.

#### 3.5.2 Timing

In allogeneic transplants, there were 4 candidemia equally prevalent in all 3 time periods posttransplant, of which all of them were caused by *Candida parapsilosis*. For mold infections, there were 1 mucormycosis detected in 31–100 days and 2 aspergillosis in 101–730 days postallogeneic transplants. For IFD in the autologous transplant, only 1 episode of *Candida* urinary tract infection was detected in 31–100 days posttransplant.

There was no significant difference comparing the frequencies of IFD between allogeneic and autologous transplants. There was also no significant difference in frequencies of IFD between the 3 time periods postallogeneic transplants.

#### 3.5.3 Risk factors

In univariate analysis, the risk of fungal infection was higher with the use of cord blood (*p* = 0.024 vs. PBSC), in patients with refractory cancer (*p* = 0.043 vs. CR1), and in patients with gut GVHD (*p* = 0.008). The occurrence of CMV pneumonia was associated with fungal infection compared with CMV viremia only (*p* = 0.018). There was no fungal infection in patients with marrow graft or in those with MSD or matched unrelated donor (MUD). In multivariate analyses, gut GVHD remained a risk factor (OR = 12.3, 95% CI = 1.33–114.4, and *p* = 0.027).

### 3.6 Infection‐related mortality

#### 3.6.1 Incidence

During the study period, 10 (13.5%) patients who had received allogeneic HSCT died of infection. Six (60%) patients died between 31 and 100 days and 4 (40%) died 100 days to 2 years post‐HSCT. For these deaths, 1 (10%) was attributed to bacteria, 3 (30%) to viruses, 5 (50%) to fungi, and 1 (10%) to mixed viral and fungal infections. No autologous transplant patient died of infection. The comorbidities present at the time of infection‐related deaths were listed in Supplemental Table S3.

#### 3.6.2 Risk factors for death from infection

**FIGURE 5:**
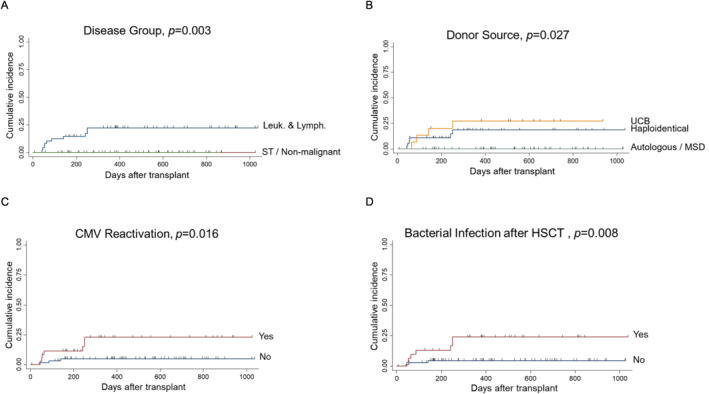
Cumulative incidences of infection‐related death. (A–D) Stratification based on various risk factors. CMV, cytomegalovirus; HSCT, hematopoietic stem cell transplantation; Leuk. and Lymph., leukemia and lymphoma; MSD, matched sibling donor; MUD, matched unrelated donor; ST, solid tumor; UCB, umbilical cord blood.

All 10 infection‐related deaths occurred in patients with hematological malignancies after receiving a cord blood (*n* = 4) or haploidentical HSCT (*n* = 6). In univariate analyses, infection‐related deaths were associated with hematological malignancies (*p* = 0.003), use of cord blood or haploidentical donor (*p* = 0.027), and history of bacterial infection (*p* = 0.008) or CMV reactivation (*p* = 0.016) after HSCT (Figure 5). In multivariate analysis, history of bacterial infection after HSCT remained a significant factor (HR = 4.13, 95% CI = 1.05–16.3, and *p* = 0.042).

## 4 DISCUSSION

IRM is the major non‐relapse cause of death after pediatric HSCT.^2^, ^26^, ^31^ In our cohort, a 10% IRM was observed, primarily due to viral and fungal infections. Furthermore, infections caused substantial morbidities, particularly in allogeneic recipients, of whom only 6.8% were infection‐free after HSCT. Similar reports on allogeneic HSCT performed one to 2 decades ago observed three to five‐fold higher infection‐free rates, which could be explained by less frequent use of cord blood, haploidentical donors, PBSCs, or ex vivo T‐cell depletion in the past.^10^, ^11^

Although bacterial infection was not a major direct cause of death, it occurred in about 30% of our patients throughout the 3 time‐periods after both autologous and allogeneic HSCT. Patients who had relapsed disease were particularly vulnerable when compared with patients in CR1. Notably, a history of bacterial infection after HSCT was the single most important prognostic factor for subsequent deaths related to viral or fungal infection. Collectively, these data suggested that relapsed patients who were heavily pretreated were susceptible to multiple sequential infections with different pathogens, supporting the judicious use of antibacterial prophylaxis to prevent the first infection event in this specific patient population,^5^ but not all others for the concern of resistance and side effects.^6^

In contrast to bacterial infections, a reverse pattern was observed for fungal infections with low incidence but high mortality, accounting for 6/10 of deaths in our cohort. These observations suggested that our practice of echinocandins prophylaxis for 1 month was useful in general, but failed to prevent many fatal fungal infections thereafter, which were attributable to poor control of comorbidities such as GVHD or regimen‐related organ failures at the time of death (Supplemental Table S3). In fact, gut GVHD was the sole risk factor for fungal infection in multivariate analysis. This finding supports continuation of antifungal prophylaxis during treatment for gut GVHD and encourages future investigations, such as prebiotics and probiotics, to restore microbiomes to reduce simultaneously the development of GVHD and fungal colonization.^32^, ^33^, ^34^

In our cohort, viral infections and reactivations were common both in incidence and in their contribution to mortality accounting for 4/10 of deaths. Three epidemiological patterns were observed. The first group, represented by HHV‐6 and BKV, had highest incidence early after HSCT, was not fatal in general, and was linked to grafts associated with slow immune reconstitution such as cord blood or ex vivo T‐cell depletion. Concurrent BKV infections with HHV‐6 were common (Figure 4H). The second pattern, observed with ADV and VZV, had disease onset late after HSCT, occurred primarily in children who were older than 10 years and were exposed to steroids after transplant. While VZV could easily be treated with acyclovir, ADV often responded poorly to cidofovir and was the primary cause of death in 2/10 of our IRM. The third group, which included potentially life‐threatening CMV and EBV, occurred commonly across all 3 time‐points, particularly in recipients of T‐cell depleted grafts in the setting of high viral load, such as hosts being seropositive for CMV or grafts containing abundant EBV‐positive B cells. In this regard, the cumulative incidence of EBV was low with cord blood and relatively low with haploidentical transplants; the latter unexpected finding was perhaps in part related to our practice of CD45RA‐depletion, which effectively removed almost all B cells and preserved memory T cells. Prior studies of CD45RA‐depletion had also found a low incidence of EBV in haploidentical HSCT.^35^ Nevertheless, these patients are at risk and should be monitored closely for a longer period until T cell immunity recovered. Concurrent reactivations of CMV and EBV were common (Supplemental Figure S2E); thus, the reactivation of one virus should prompt close monitoring of the other.

**FIGURE 6:**
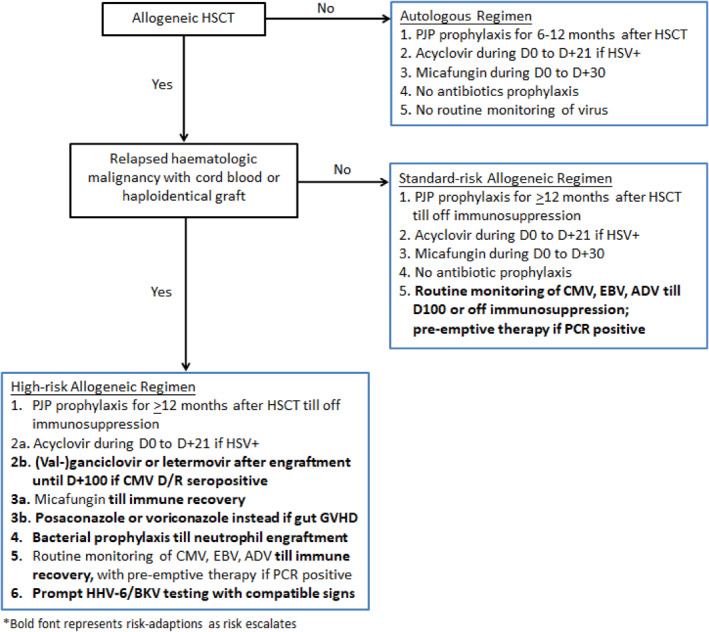
Revised algorithm for prophylactic antimicrobials and infection monitoring. ADV, adenovirus; BKV, BK virus; CMV, cytomegalovirus; CMV D/R, cytomegalovirus donor/recipient; EBV, Epstein–Barr virus; GVHD, graft‐versus‐host disease; HHV‐6, human herpesvirus 6; HSCT, hematopoietic stem cell transplantation; HSV, herpes simplex virus; PCR, polymerase chain reaction; PJP, *Pneumocystis jirovecii* pneumonia.

An important finding of the study is that only patients with hematological malignancies who underwent cord blood or haploidentical HSCT died from infection. Currently, most recommendations guide prophylaxis according to the type of transplantation (allogeneic/autologous) and the phase of immune reconstitution. Based on our new findings, stratification of infection prophylaxis according to the primary disease and donor type was optimized in our center (Figure 6). This vulnerable group of patients should be monitored closely, especially if they have a history of bacterial or CMV infection after HSCT as suggested by our risk factor analysis, which are surrogate markers of poor innate and adaptive immune functions. Intensive preemptive antiviral therapy and antifungal prophylaxis, where viral and fungal infections were the major causes of death, might be warranted in this ultra‐high risk group.^7^, ^8^, ^9^ Good control of CMV could in turn reduce the risk of GVHD and fungal infection.^36^, ^37^ Latest guidelines^3^, ^7^ also recommend the use of the newer triazole group, for example, posaconazole and voriconazole, as primary prophylaxis.

Our study has several strengths and weaknesses. First, selection bias was avoided by inclusion of all consecutive patients since the opening of the Children's Hospital, which is the only hospital that provides pediatric HSCT services in our entire territory since April 2019. Second, hazard functions were analyzed for viral infections, taking into account the time of onset. Even if the incidences are the same, earlier reactivation of a particular virus often indicates more profound immunodeficiency and more severe disease, requiring longer treatment with higher risk of side effects. Thus, assessing hazard functions rather than relative odds might be more informative and statistically efficient. As for the limitations of our study, it is a retrospective study without specified hypotheses a priori. Furthermore, GVHD was defined clinically without measurement of discerning biomarkers.^38^ The severity of infections was not classified except fatal versus nonfatal, and their economic costs were not assessed.^39^, ^40^

In conclusion, our study has identified the epidemiology and risk factors for bacterial, viral, and fungal infections after contemporary HSCT. These recent findings should be beneficial in optimization of personalized strategies for infection prophylaxis and monitoring.

## AUTHOR CONTRIBUTIONS

Tsz Wing Yeung and Wing Leung designed the study. Tsz Wing Yeung, Wilson Yau Ki Chan, Pamela Pui Wah Lee, and Daniel Ka Leung Cheuk collected the data. Tsz Wing Yeung and Wing Leung analyzed the data and drafted the manuscript. Sally Cheuk Ying Wong gave conceptual advice. All authors read, edited, and approved the final manuscript.

## CONFLICT OF INTEREST STATEMENT

The authors declare they have no conflicts of interest.

## ETHICS STATEMENT

This study was approved by the Research Ethics Committee of the Hong Kong Children's Hospital.

## Supporting information

Supplementary Material

## Data Availability

Research data are not shared.
