# Correction to High Infection Rates and Risk‐Adapted Prevention Strategies in Contemporary Pediatric Allogeneic Hematopoietic Stem Cell Transplantation

**DOI:** 10.1002/pdi3.2527

**Published:** 2025-03-05

Yeung TW, Chan WYK, Wong SCY, Lee PPW, Cheuk DKL, Leung W. High infection rates and risk‐adapted prevention strategies in contemporary pediatric allogeneic hematopoietic stem cell transplantation. *Pediatr Discov*. 2024;2(4):e101. https://doi.org/10.1002/pdi3.101

The affiliations of three authors need to be corrected.

[1. Department of Pediatrics and Adolescent Medicine, Hong Kong Children's Hospital, Kowloon, Hong Kong.

2. Department of Pathology, Hong Kong Children's Hospital, Kowloon, Hong Kong.

3. Department of Pediatrics and Adolescent Medicine, Queen Mary Hospital, Li Ka Shing Faculty of Medicine, The University of Hong Kong, Pok FuLam, Hong Kong].

According to the policy of our journal, “Hong Kong” can only be expressed as “Hong Kong SAR, China”. The former affiliations had been replaced with the following new affiliations:

1. Department of Paediatrics and Adolescent Medicine, Hong Kong Children's Hospital, Kowloon, Hong Kong SAR, China.

2. Department of Pathology, Hong Kong Children's Hospital, Kowloon, Hong Kong SAR, China.

3. Department of Paediatrics and Adolescent Medicine, Queen Mary Hospital, Li Ka Shing Faculty of Medicine, The University of Hong Kong, Pok Fu Lam, Hong Kong SAR, China.

We apologize for this error.

