# Supplementary material for: High infection rates and risk‐adapted prevention strategies in contemporary pediatric allogeneic hematopoietic stem cell transplantation
Source: Pediatr Discov. 2024 Jul 14;2(4):e101. doi: 10.1002/pdi3.101 (PMC12118308; doi:10.1002/pdi3.101)
Supplement: Supplementary file 1 — Supplementary Material [file PDI3-2-e101-s001.docx]

**SUPPLEMENTAL TABLE 1** Pathogens of bacteremia 0-30 days, 31-100 days, 101-730 days post-transplant

**SUPPLEMENTAL TABLE 2** Viral infections on 0-30 days, 31-100 days, and 101-730 days post-allogeneic transplant

**SUPPLEMENTAL TABLE 3** Infection-related deaths and comorbidities

**SUPPLEMENTAL FIGURE 1** Cumulative incidences of cytomegalovirus

Abbreviations: CMV D/R, cytomegalovirus donor/recipient; CR1, first complete remission; CR2, second complete remission; CR3, third complete remission; MSD, matched sibling donor; MUD, matched unrelated donor; UCB, umbilical cord blood.

**SUPPLEMENTAL FIGURE 2** Cumulative incidences of Epstein-Barr virus

Abbreviations: CMV, cytomegalovirus; MSD, matched sibling donor; MUD, matched unrelated donor; TB, total body irradiation; UCB, umbilical cord blood.

**SUPPLEMENTAL TABLE 1** Pathogens of bacteremia in 0-30 days, 31-100 days, 101-730 days post-transplant

| Pathogens | Allogeneic | | | | Autologous | | | |
| --- | --- | --- | --- | --- | --- | --- | --- | --- |
|  | 0-30 days | 31-100 days | 101-730 days | **Total**  **episodes** | 0-30 days | 31-100 days | 101-730 days | **Total episodes** |
| **GP bacteria** | 6 | 2 | 2 | **10** | 0 | 0 | 1 | **1** |
| *Staphylococcus aureus* | 0 | 0 | 0 | 0 | 0 | 0 | 1 | 1 |
| CoNS | 4 | 2 | 1 | 7 | 0 | 0 | 0 | 0 |
| *Staphylococcus hemolyticus* | 1 | 0 | 0 | 1 | 0 | 0 | 0 | 0 |
| *Staphylococcus epidermidis** | 3 | 2 | 1 | 6 | 0 | 0 | 0 | 0 |
| *Abiotrophia defectiva* | 1 | 0 | 0 | 1 | 0 | 0 | 0 | 0 |
| *Streptococcus mitis* | 1 | 0 | 0 | 1 | 0 | 0 | 0 | 0 |
| *Granulicatella adiacens* | 0 | 0 | 1 | 1 | 0 | 0 | 0 | 0 |
| **GN bacteria** | 7 | 3 | 2 | **12** | 1 | 1 | 1^Δ^ | **3**^Δ^ |
| *Pseudomonas aeruginosa** | 3 | 0 | 1 | 4 | 0 | 1 | 0 | 1 |
| *Pseudomonas mendocina* | 0 | 0 | 0 | 0 | 0 | 0 | 1^Δ^ | 1^Δ^ |
| *Enterobacter cloacae** | 1 | 2 | 1 | 4 | 0 | 0 | 0 | 0 |
| *Escherichia coli* | 1 | 0 | 0 | 1 | 0 | 0 | 0 | 0 |
| *Klebsiella pneumoniae* | 2 | 0 | 0 | 2 | 1 | 0 | 0 | 1 |
| *Stenotrophomonas maltophilia* | 0 | 1 | 0 | 1 | 0 | 0 | 1^Δ^ | 1^Δ^ |
| *Acinetobacter* species | 0 | 0 | 0 | 0 | 0 | 0 | 1^Δ^ | 1^Δ^ |
| **Total episodes** | 13 | 5 | 4 | **22** | 1 | 1 | 2 | **4** |

CoNS, coagulase-negative *Staphylococcus*; GP, Gram-positive; GN, Gram-negative.

*3 allogeneic transplant patients had recurrence of bacteremia (*S. epidermidis; Enterobacter cloacae; Pseudomonas aeruginosa*).

Δ One autologous transplant patient had mixed growth in blood culture with *Pseudomonas mendocina, Acinetobacter species, Stenotrophomonas maltophilia.*

**SUPPLEMENTAL TABLE 2** Viral infections on 0-30 days, 31-100 days, and 101-730 days post-allogeneic transplant

|  | 0-30 days,  n=74 (%) | 31-100 days,  n=72 (%) | 101-730 days,  n=58 (%) | *P* value |
| --- | --- | --- | --- | --- |
| **Viral infections** |  |  |  |  |
| **Any viral infection** | 53 (71.6) | 43 (59.7) | 31 (53.4) | 0.078 |
| **CMV** | 24 (32.4) | 16 (22.2) | 15 (25.9) | 0.371 |
| **ADV** | 1 (1.4) | 10 (13.9) | 6 (10.3) | 0.019 |
| **EBV** | 17 (23.0) | 22 (30.6) | 14 (24.1) | 0.745 |
| **HHV-6** | 26 (35.1) | 5 (6.9) | 5 (8.6) | <0.001 |
| **HHV-7** | 0 (0) | 0 (0) | 0 (0) | - |
| **BKV** | 22 (29.7) | 15 (20.8) | 2 (3.4) | 0.001 |
| **HSV** | 0 (0) | 1 (1.4) | 1 (1.7) | 0.552 |
| **VZV** | 0 (0) | 0 (0) | 5 (8.6) | 0.002 |
| **Other viruses** | 0 (0) | 1 (1.4) ^δ^ | 4 (6.9) ^θ^ | 0.030 |

ADV, adenovirus; BKV, BK virus; CMV, cytomegalovirus; EBV, Epstein-Barr virus; HHV-6, human herpesvirus 6; HHV-7, human herpesvirus 7; HSV, herpes simplex virus; VZV, varicella zoster virus.

δ Norovirus.

θ Rat hepatitis E virus, respiratory syncytial virus, norovirus, and rhinovirus/enterovirus.

**SUPPLEMENTAL TABLE 3** Infection-related deaths and comorbidities

| No. | Causes of Infection-related deaths | Types of pathogen | Comorbidities, if any | Day of death  post-HSCT |
| --- | --- | --- | --- | --- |
| 1 | Pulmonary aspergillosis | Fungus | GVHD, respiratory failure | D+242 |
| 2 | Pulmonary aspergillosis | Fungus | GVHD | D+251 |
| 3 | CMV pneumonia | Virus |  | D+42 |
| 4 | Disseminated candidiasis | Fungus | Multi-organ failure | D+87 |
| 5 | Disseminated candidiasis | Fungus | Renal failure, GVHD | D+141 |
| 6 | CMV pneumonia | Virus |  | D+64 |
| 7 | Adenovirus pneumonia and candidemia | Virus & Fungus | Multi-organ failure, graft failure | D+46 |
| 8 | Pseudomonas septicemia and necrotizing fasciitis | Bacteria | Disease progression | D+251 |
| 9 | Invasive mucormycosis | Fungus | TEN, GVHD, GI bleeding, hyperbilirubinemia | D+53 |
| 10 | Adenovirus pneumonia | Virus |  | D+55 |

CMV, Cytomegalovirus; GI, Gastrointestinal; GVHD, graft-versus-host disease; TEN, Toxic epidermal necrolysis.

**SUPPLEMENTAL FIGURE 1** Cumulative incidences of cytomegalovirus

| A  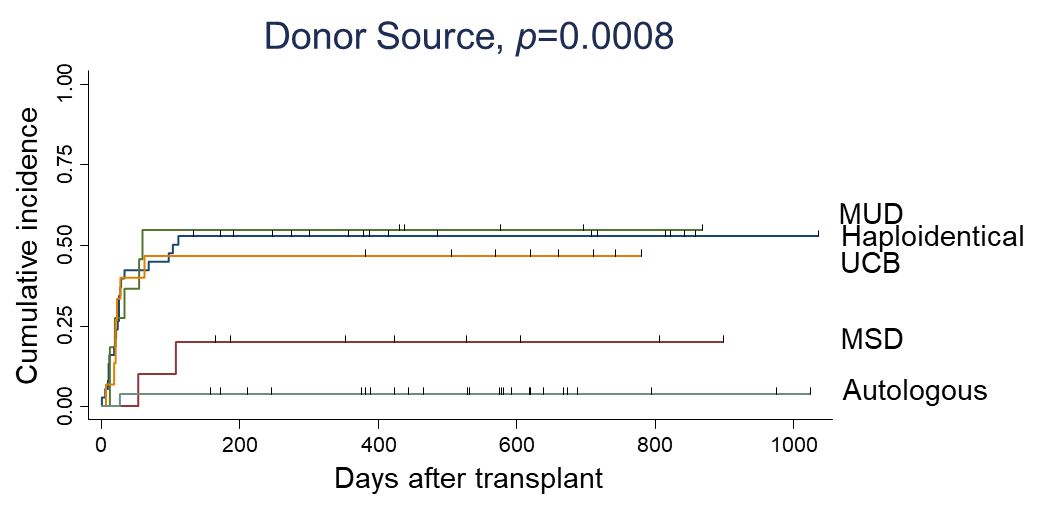 | B  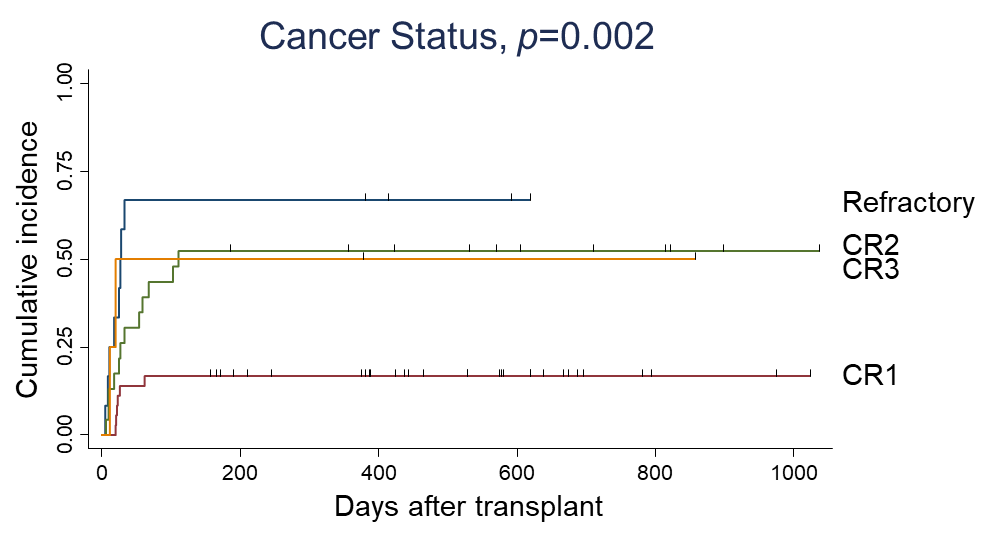 |
| --- | --- |
| C  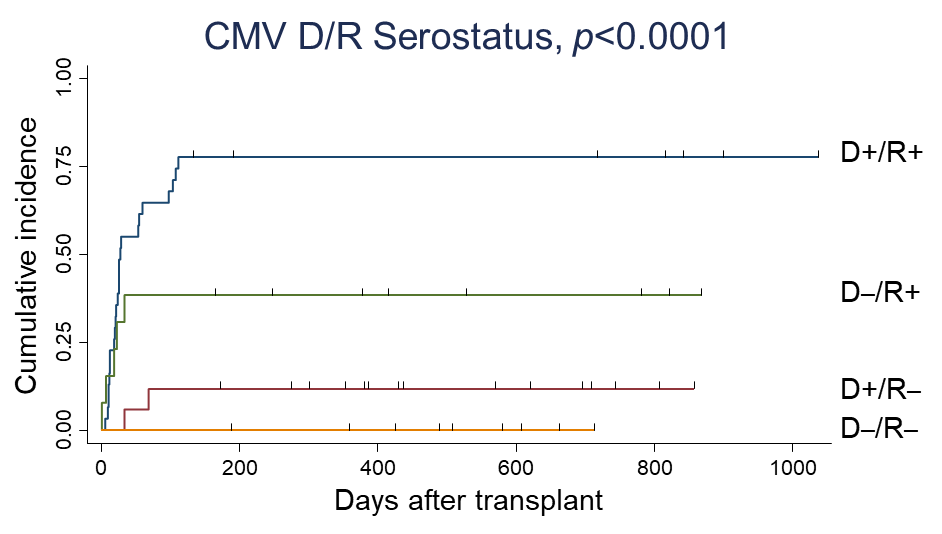 | 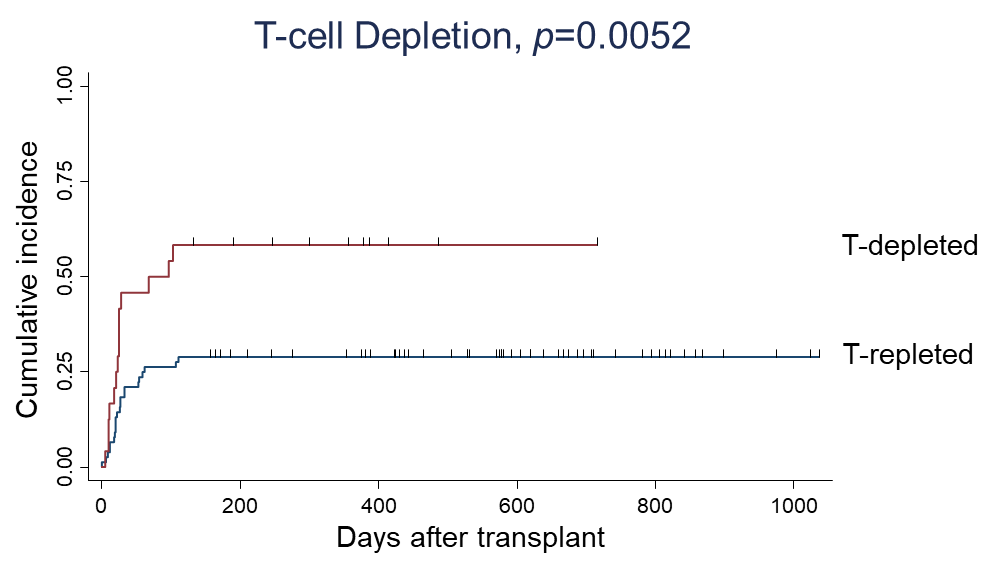D |
| 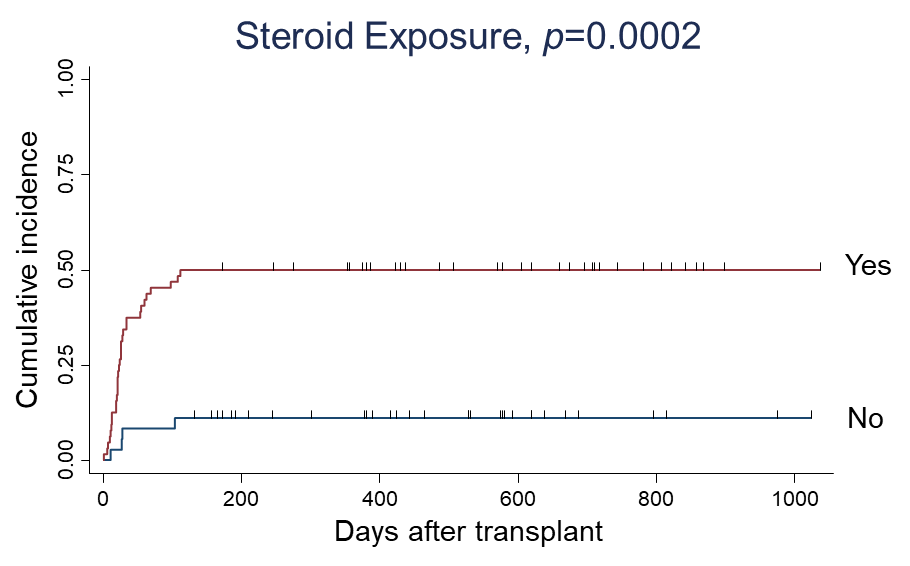E |  |

CMV D/R, cytomegalovirus donor/recipient; CR1, first complete remission; CR2, second complete remission; CR3, third complete remission; MSD, matched sibling donor; MUD, matched unrelated donor; UCB, umbilical cord blood.

**SUPPLEMENTAL FIGURE 2** Cumulative incidences of Epstein-Barr virus

| A  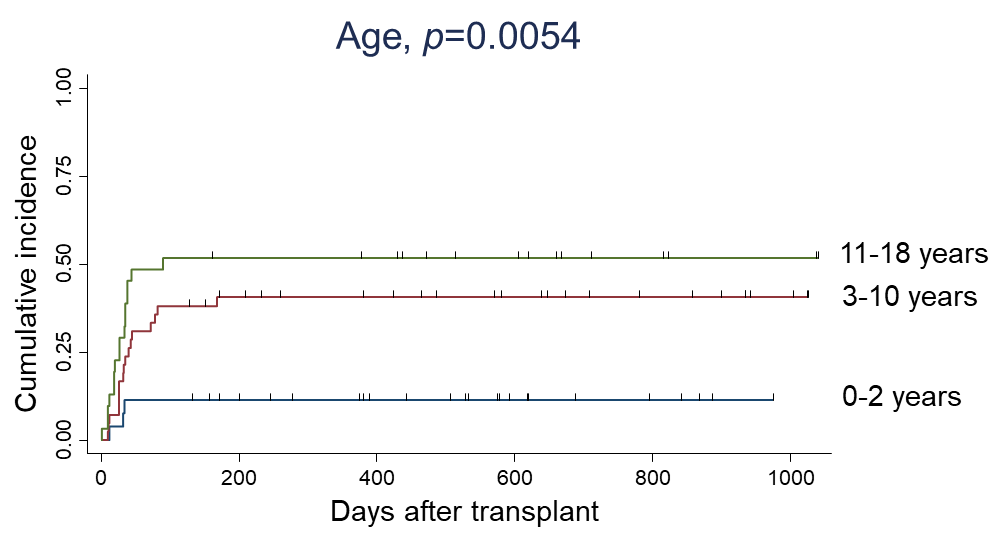 | B  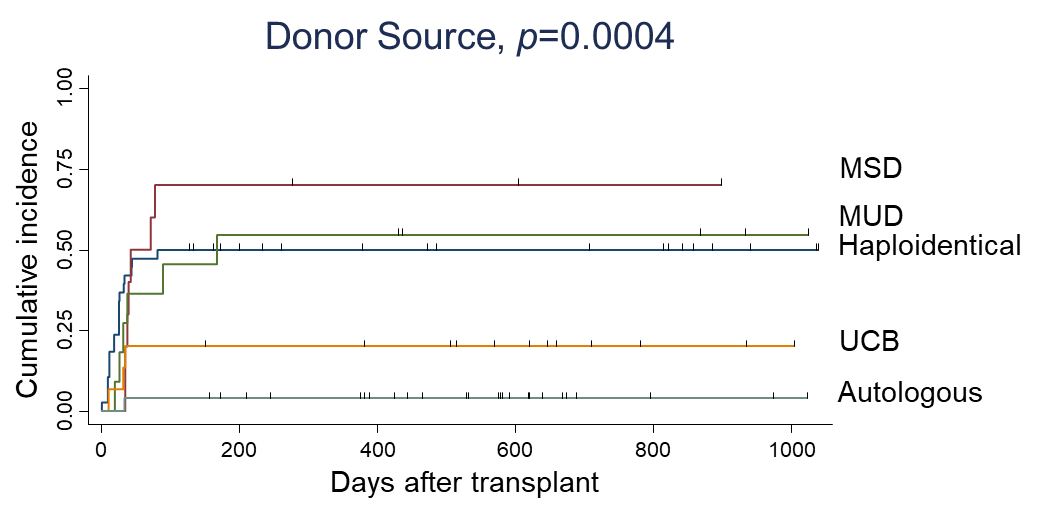 |
| --- | --- |
| C  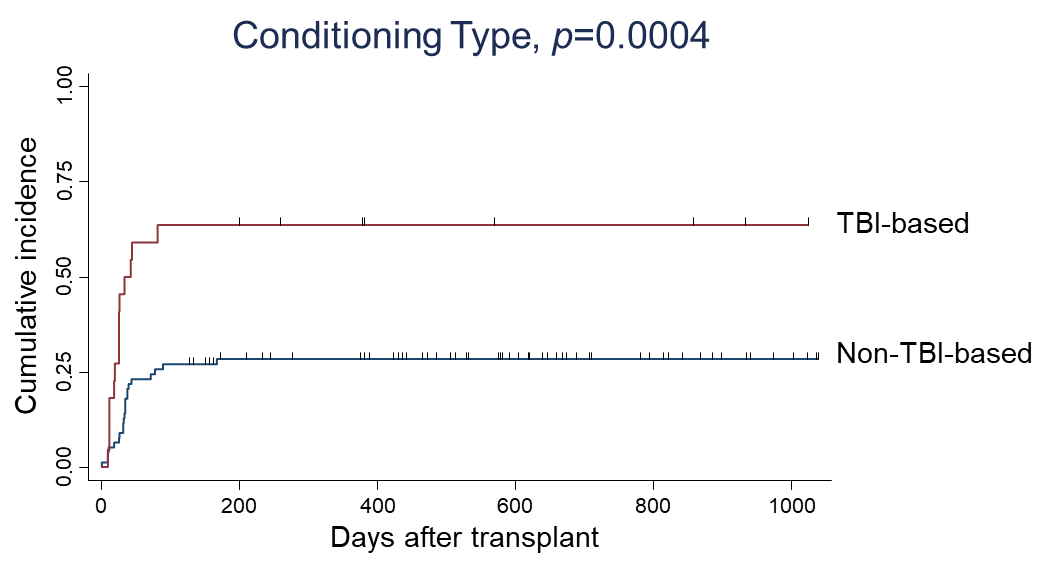 | D  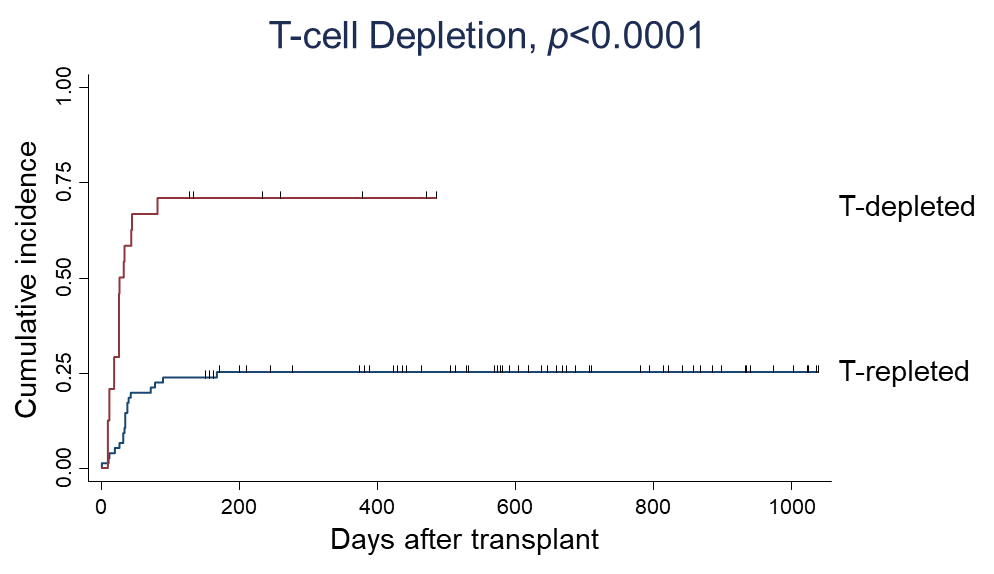 |
| E  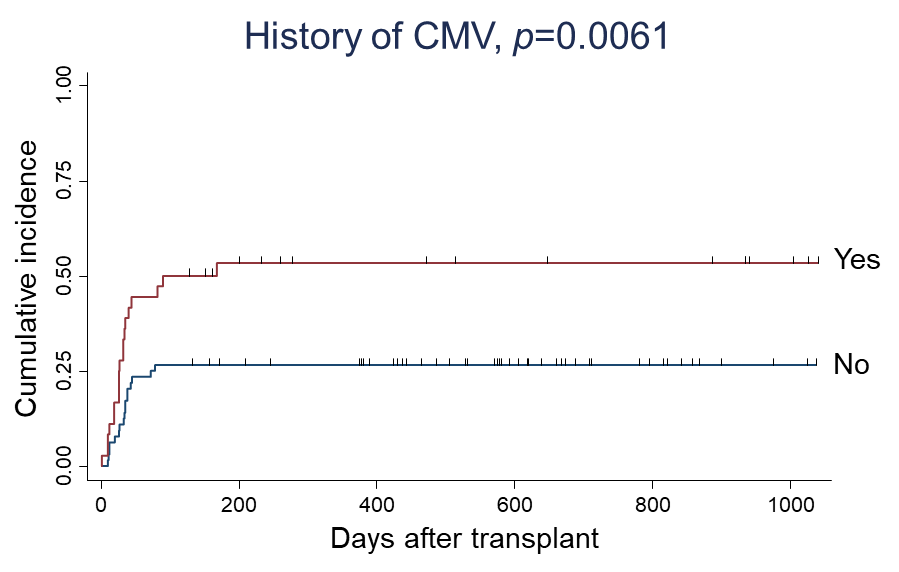 |  |

CMV, cytomegalovirus; MSD, matched sibling donor; MUD, matched unrelated donor; TB, total body irradiation; UCB, umbilical cord blood.
